# MYEOV functions as an amplified competing endogenous RNA in promoting metastasis by activating TGF-β pathway in NSCLC

**DOI:** 10.1038/s41388-018-0484-9

**Published:** 2018-09-04

**Authors:** Lishan Fang, Shanshan Wu, Xun Zhu, Junchao Cai, Jueheng Wu, Zhenjian He, Lei Liu, Musheng Zeng, Erwei Song, Jun Li, Mengfeng Li, Hongyu Guan

**Affiliations:** 1 Key Laboratory of Tropical Disease Control (Sun Yat-sen University), Ministry of Education, Guangzhou, China; 2 Central Laboratory of The Eighth Affiliated Hospital, Sun Yat-sen University, Shenzhen, China; 3 Department of Microbiology, Zhongshan School of Medicine, Sun Yat-sen University, Guangzhou, China; 4 Guangdong Provincial Key Laboratory of Orthopedics and Traumatology, Guangzhou, China; 5 School of Public Health, Sun Yat-sen University, Guangzhou, China; 6 State Key Laboratory of Oncology in South China, Department of Experimental Research, Sun Yat-Sen University Cancer Center, Guangzhou, China; 7 Department of Breast Surgery, Sun Yat-Sen Memorial Hospital, Sun Yat-Sen University, Guangzhou, China; 8 Department of Biochemistry, Zhongshan School of Medicine, Sun Yat-sen University, Guangzhou, China; 9 Department of Endocrinology, The First Affiliated Hospital of Sun Yat-sen University, Guangzhou, China

**Keywords:** Metastasis, Cell signalling, Non-small-cell lung cancer, Metastasis, Cell signalling

## Abstract

Non-small cell lung cancer (NSCLC) remains a major cause of death worldwide. As metastatic disease is primarily responsible for the poor clinical outcome of NSCLC, it is important to understand the process, and its underlying molecular mechanism as well, via which NSCLC cells disseminate. In this study, we identified a new competing endogenous RNA (ceRNA), namely, the MYEOV transcript, and found that it is upregulated in NSCLC and associated with a poor prognosis of the disease. We further uncovered that the MYEOV ceRNA plays a critical role in the invasion and metastasis of NSCLC cells. Intriguingly, the MYEOV ceRNA exerted its pro-metastatic function independent of its protein-coding capacity, but in a miR-30c-2-3p binding-dependent manner. Further experiments demonstrated that the MYEOV ceRNA sequestered miR-30c-2-3p from binding its targets TGFBR2 and USP15 mRNAs, which in turn leading to constitutive activation of TGF-β signaling and tumor progression in NSCLC. By identifying a new layer of regulatory modality for TGF-β signaling, our findings extend the current understanding on the molecular mechanism mediating NSCLC progression and highlight a potential role of MYEOV transcript to serve as the therapeutic target.

## Introduction

Lung cancer remains a major cause of death worldwide, and non-small cell lung cancer (NSCLC) accounts for at least 80% of all lung cancer cases diagnosed [1]. Despite the advances made over the past decades in the treatment of NSCLC, the overall 5-year survival rate of the disease remains lower than 15% for all stages combined [2]. Metastatic disease is primarily responsible for the generally low survival of NSCLC, and therefore, better understanding of the molecular mechanisms via which NSCLC disseminate is needed [3, 4]. Identifying novel molecules that can repress the invasiveness and metastasis of lung cancer will facilitate the development of new anti-lung cancer strategies.

It is well established that transforming growth factor-β (TGF-β) triggered signaling plays an instrumental role in activating the biological process of tumor invasion and metastasis [5–7]. In canonical SMAD-dependent signaling, TGF-β binds directly to TGF-β receptor type II (TGFBR2) and subsequently recruits and phosphorylates TGFBR1, leading to activation and translocation of SMADs to regulate downstream genes essential for metastasis [8]. Strikingly, dysregulated activation of TGF-β signaling and the abovementioned components of the pathway have been found in the process of NSCLC progression [9, 10]. Nevertheless, unlike in other cancer types such as colon cancer, mutations in TGFBR2 and SMAD2/4 genes appear to be uncommon in NSCLC, according to previously reported studies [11, 12] and analysis of the catalog of somatic mutations in cancer (COSMIC), suggesting that additional mechanisms involved in maintaining constitutive activation of TGF-β signaling cascades are to be revealed in NSCLC.

Myeloma overexpressed gene (MYEOV) locates in chromosome (chr)11q13.3, a region of cancer-associated genomic amplification [13–16], has been reported to be predominantly overexpressed and contribute to tumorigenesis in many human cancer types, including multiple myeloma [17], neuroblastoma [18], esophageal squamous cell carcinoma [19], breast cancer [20], gastric cancer [21], and colon cancer [22]. While dysregulated expression of MYEOV transcript in cancer patients has been associated with its tumorigenic properties, the molecular mechanisms underlying MYEOV-mediated tumorigenesis are still largely unclear. Study by Horie et al. predicted Myc as the most affected transcriptional factor in response to knockdown of MYEOV using Integrated Motif Activity Response Analysis (ISMARA) [23]. The finding that 17 out of 38 MYEOV depletion-downregulated genes carry Myc binding sites within the promoter regions supports the prediction, suggesting an involvement of Myc transcriptional activity in MYEOV-mediated tumorigenesis [23]. Intriguingly, previous studies have identified a mismatched expression pattern between MYEOV transcript and its protein, as dramatically highly-expressed MYEOV transcript in cancer cell cannot be translated to any mature protein and the biosynthesis of MYEOV protein can be suppressed by its own 5′-UTR sequence [24], indicating that additional functions rather than protein-coding capacity for MYEOV transcript may exert its observed role in tumorigenesis.

Recently, compelling evidence supports that a class of RNA molecules, such as mRNA [25], transcribed pseudogenes [26], long noncoding RNAs [27], and circular RNAs [28], could function as competing endogenous RNAs (ceRNA), which are able to compete against binding of a specific microRNA (miRNA) to the miRNA recognition elements (MREs) on its target mRNA, thereby serving as a “miRNA sponge” to efficiently de-repress the target mRNA [29, 30]. Such a proposed ceRNA theory provides a new mechanistic basis for understanding the pro-metastatic role of MYEOV transcript in NSCLC.

In this study, we found that frequently amplificated MYEOV was significantly upregulated at the transcriptional level and associated with prognosis of patients in NSCLC. Independent of its protein-coding capacity, MYEOV transcript exerts its pro-metastatic function via abrogating the suppression of TGFBR2 and Ubiquitin specific protease 15 (USP15) expression by miR-30c-2-3p, leading to constitutive activation of TGF-β signaling and tumor progression in NSCLC. These findings illuminated novel mechanism of MYEOV transcript and approach of TGF-β activation via ceRNA mediated network, highlighting a potential role of MYEOV transcript to serve as the therapeutic target.

## Results

### Amplification of MYEOV gene locus and its upregulated expression in NSCLC

**Fig. 1:**
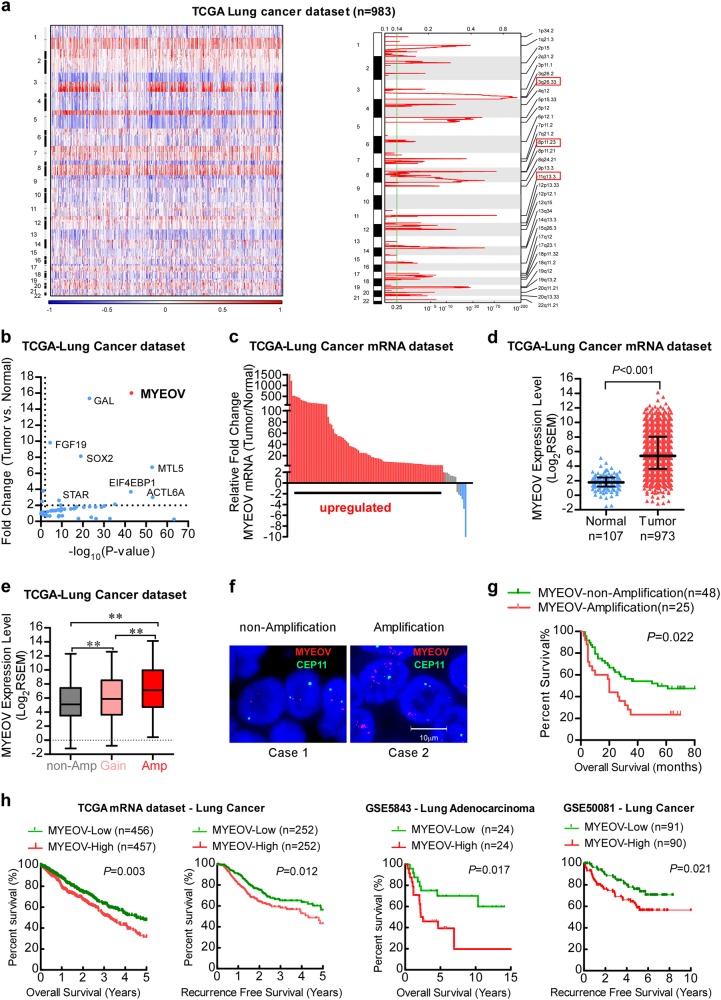
Amplification and upregulation of MYEOV transcript in NSCLC. **a** Copy number data for 983 lung cancer samples are shown with genomic locations. The color scale ranges from blue (deletion) through white (neutral; diploid) to red (amplification). False discovery rates (*q* values; green line is 0.25 cutoff for significance) and scores (log_2_ ratio ≥ 0.8) for amplicons are labeled at each genome position; red boxes indicate the top three significant high-level amplifications. **b** Fold changes of mRNA expression and statistic *P*-value when comparing NSCLC and noncancerous adjacent tissue on the indicated genes within the amplicon. **c** Statistical analysis of MYEOV expression in NSCLC and adjacent noncancerous tissue in the TCGA cohort. **d** MYEOV mRNA expression in 973 NSCLC specimens and 107 normal lung specimens in the TCGA dataset. Horizontal lines represent the means, and each dot represents an individual specimen (median with interquartile range, two-tailed unpaired Student’s *t* test). **e** Relationship between the CNVs of MYEOV and its mRNA expression level in the TCGA cohort. Box plot represents lower quartile; median and upper quartile and whiskers represent 95% confidence interval of the mean (one-way ANOVA followed by Bonferroni’s multiple comparison test. **P < 0.01). **f** MYEOV genomic amplification status confirmed by FISH assay. Red signals indicate the MYEOV, and green signals represent CEP11. **g** Kaplan–Meier survival analysis of NSCLC patients in the SYSUCC cohort stratified by MYEOV genomic amplification status. **h** Kaplan–Meier analyses of the correlations between MYEOV expression level and overall survival or recurrence-free survival of NSCLC patients in TCGA dataset and in GSE5843 and GSE50081, respectively. The median expression level was used as the cutoff with the *P* value of log-rank test presented for each set

To gain insight into the genetic changes at the genomic level in NSCLC, we performed a whole-genome copy number variation (CNV) analysis of 493 lung adenocarcinoma and 490 lung squamous cell carcinoma cases in The Cancer Genome Atlas (TCGA) dataset. As shown in Fig. 1a, we found that regions of amplification distributed widely across the whole genome and the most frequently observed regions of amplification were chr3q26.33, chr11q13.3 and chr8p11.23. Notably, when we attempted to further identify target genes for aforementioned amplicons by analyzing RNA-seq data in the primary tumors and adjacent non-cancerous tissues, MYEOV emerged as the most strikingly overexpressed gene (Fig. 1b; Supplementary Figure S1a). As shown in Fig. 1c, 92 out of 107 pairs (86.0%) of TCGA samples tested showed greater than two-fold increase of MYEOV mRNA level in tumor lesions as compared with that in the matched adjacent non-cancerous tissue. Moreover, MYEOV mRNA was significantly upregulated in NSCLC tissues (*n* = 973) compared with adjacent non-cancerous lung tissues (*n* = 107) in TCGA cohort (Fig. 1d). Indeed, chr11q13.3 and MYEOV amplification status are also confirmed in the Cancer Cell Line Encyclopedia (CCLE)—lung cancer line dataset and the Tumorscape-NSCLC dataset (Supplementary Figure S1b). In addition, mRNA expression level of MYEOV is positively correlated with its amplification status (Fig. 1e). Furthermore, we conducted fluorescence in situ hybridization assay (FISH) to analyze MYEOV copy number variations in our samples (SYSUCC cohort, *n* = 73) (Fig. 1f) and the survival curves revealed that patients with MYEOV amplification had poorer overall survival (OS) than those without MYEOV amplification (log rank test, *P* = 0.022, Fig. 1g). In addition, the amplification status of MYEOV significantly correlated with high TNM clinical staging (*P* = 0.002), lymph nodes metastasis (*P* = 0.032), and metastasis status (*P* = 0.036) (Supplementary Table S1). Consistently, quantitative reverse transcription-polymerase chain reaction (qRT-PCR) assay further confirmed that MYEOV transcript was significantly overexpressed in NSCLC tissues and positively correlated with its amplification status (Supplementary Figure S1c and S1d). We further examined whether the MYEOV expression was associated with the clinical outcome of NSCLC patients using publicly available datasets. As shown in Fig. 1h, Kaplan-Meier analysis in the patients with NSCLC from TCGA data set revealed that high MYEOV expression level in NSCLC tissues significantly correlated with a reduction in OS (*P* = 0.003) and recurrence-free survival (*P* = 0.012). Moreover, our COX analysis revealed a significant prognostic power of MYEOV as a marker of poor clinical outcome (Supplementary Table S2, Cox hazard ratio: 1.618; 95% CI 1.213–2.159). To extend and validate these findings, we performed similar survival analyses of MYEOV in other independent clinical array panels of NSCLC cohorts (GSE5843 and GSE50081) and obtained similar results (Fig. 1h). These results together indicate that MYEOV gene is amplified and overexpressed in NSCLC and MYEOV amplification predicts poor prognosis of NSCLC patients.

Next, we assessed the DNA copy number and mRNA expression level of MYEOV in NSCLC cell lines. As shown in Supplementary Figure S1e, 6 of 12 (50%) NSCLC cell lines possessed MYEOV copy number amplification and a significant correlation between MYEOV copy number and its mRNA expression level was also observed, suggesting that the upregulation of MYEOV expression in NSCLC cells may be attributed to amplification of its gene as a significant component of the 11q13.3 amplicon.

Interestingly, analysis of the TCGA data sets revealed MYEOV copy number amplification was increased in subtypes of a number of tumor types, including esophageal squamous cell carcinoma, head and neck squamous cell carcinoma, ovarian serous cystadenocarcinoma, breast cancer, bladder carcinoma and gastric adenocarcinoma (Supplementary Figure S2a). Moreover, the levels of MYEOV mRNA were significantly upregulated, and a positive correlation between MYEOV mRNA levels and MYEOV copy number, was observed in bladder carcinoma and gastric adenocarcinoma (Supplementary Figure S2b and S2c). Taken together, these results suggest that amplification and overexpression of MYEOV gene may be an event frequently occurring in different types of cancers.

### Characterization of MYEOV transcript as a ceRNA in NSCLC

**Fig. 2:**
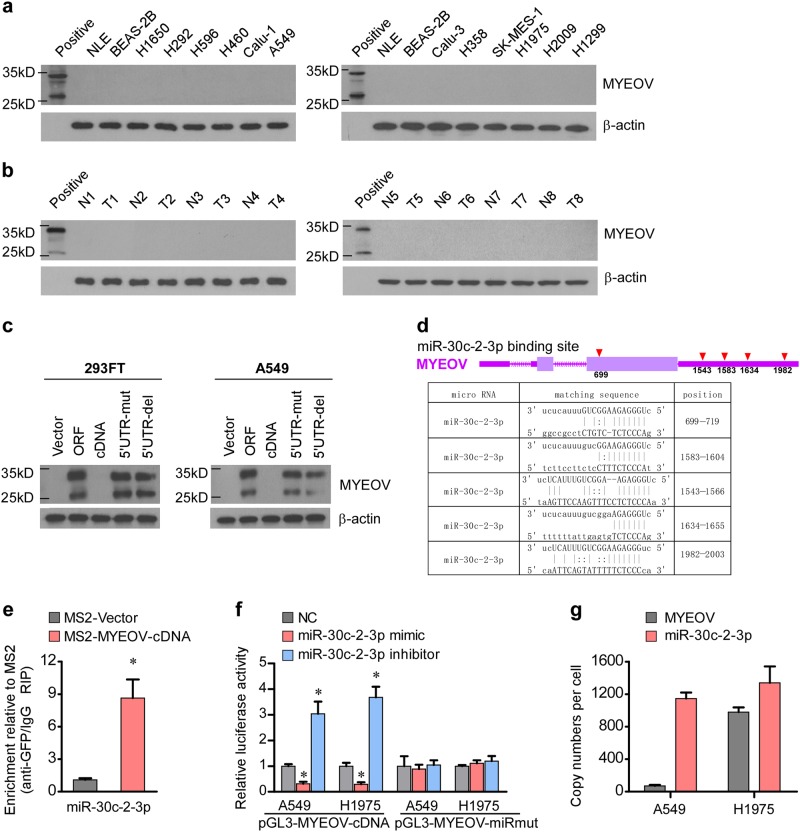
Characterization of MYEOV transcript as a ceRNA in NSCLC. **a** The protein expression levels of MYEOV in indicated cells examined by WB analysis. **b** The protein expression levels of MYEOV in NSCLC versus the paired adjacent noncancerous tissue examined by WB analysis. **c** The protein expression levels of MYEOV in indicated cells assessed by WB analysis. **d** Schematic outlining of the predicted binding sites of miR-30c-2-3p in MYEOV transcript. **e** MS2-RIP followed by miRNA qRT-PCR to detect miR-30c-2-3p endogenously associated with MYEOV transcript (each bar represents the mean ± SD derived from three independent experiments, two-tailed Student’s *t* test. *P < 0.05). **f** The reporters containing WT or mutant MYEOV transcript were co-transfected with miR-30c-2-3p mimic or inhibitor, and luciferase activities were assessed after 48 h (each bar represents the mean ± SD derived from three independent experiments, one-way ANOVA followed by Dunnett’s multiple comparison test. *P < 0.05). **g** Copy numbers of the MYEOV transcript and miR-30c-2-3p in NSCLC cell lines quantified by absolute quantitative PCR (each bar represents the mean ± SD derived from three independent experiments)

The upregulation of MYEOV transcript prompted us to further investigate the protein expression level of MYEOV in NSCLC. Surprisingly, our results showed that protein expression of MYEOV was not detectable in either NSCLC cell lines or tissue samples, whereas the antibody used in the analysis could indeed detect purified recombinant MYEOV protein (Fig. 2a, b), suggesting that there might be a suppressive mechanism that acted to abrogate the translation of the MYEOV gene. Interestingly, a previous report also has shown that although MYEOV gene contains a conventional open reading frame, translation of the gene was suppressed by its upstream AUG triplets in the MYEOV-5’UTR [24]. In following up with this notion, we further examined whether mutations of the AUG triplets in the 5′UTR of MYEOV transcript could restore the translation of MYEOV transcript. Intriguingly, our results demonstrated that the absent MYEOV translation was indeed associated with the MYEOV-5’UTR sequence in 293FT and A549 cell lines (Fig. 2c). Collectively, our results provided evidence that MYEOV transcript was robustly overexpressed without being translated into a protein product, suggesting that the MYEOV transcript might act as a functional RNA molecule in cells.

Next, to reveal the transcription start as well as terminator sites of the MYEOV transcript, 5’RACE and 3’RACE assays were performed and the results are shown in Supplementary Figure S3a–c. We hypothesized that MYEOV may function as a ceRNA and potential MREs on MYEOV were searched using miRanda v3.3a [31]. Given that the number of competing binding sites present in the transcript may affect the activity of miRNAs [32], miRNAs (miR-30c-2-3p, miR-149-3p, miR-765, and miR-3614-5p) which have five or more competing binding sites in MYEOV transcript were selected for analyses (Fig. 2d and Supplementary Figure S4a). RNA immunoprecipitation (RIP) assay was performed using the MS2-binding protein (MS2BP) system, in which tagged MS2BP specifically binds RNA containing MS2-binding sequences. We found that while little (about 2-folds for miR-149-3p) or no (for miR-765 and miR-3614-5p) significant enrichment in MS2-MYEOV pulldown products was observed, miR-30c-2-3p was the most significantly enriched (about 9-folds) in MS2-MYEOV pulldown products as compared with that in the control with the empty vector (MS2-Vector) (Fig. 2e and Supplementary Figure S4b) and therefore chose it for further investigation. Furthermore, we also found that transfection with miR-30c-2-3p mimic significantly reduced, but miR-30c-2-3p inhibitor significantly enhanced, the luciferase activity of the reporter construct containing a complete wild-type MYEOV transcript, whereas the miR-30c-2-3p mimic and the inhibitor failed to influence the reporter luciferase activity derived from the MYEOV-miRmut (point mutations in miR-30c-2-3p binding sites) (Fig. 2f), indicating that miR-30c-2-3p is indeed a direct binding partner to MYEOV transcript. In addition, we employed absolute quantitative PCR to quantify the exact copy numbers of MYEOV and miR-30c-2-3p. As shown in Fig. 2g, endogenous expression level of MYEOV was approximately 70 copies per cell in A549 and 1000 copies per cell in H1975, and the copy number of miR-30c-2-3p was approximately 1140 copies per cell in A549 and 1300 copies per cell in H1975. These results support the hypothesis that MYEOV transcript may serve as a ceRNA to bind miR-30c-2-3p.

### MYEOV transcript enhances invasion and metastasis of NSCLC in a miR-30c-2-3p binding-dependent manner

**Fig. 3:**
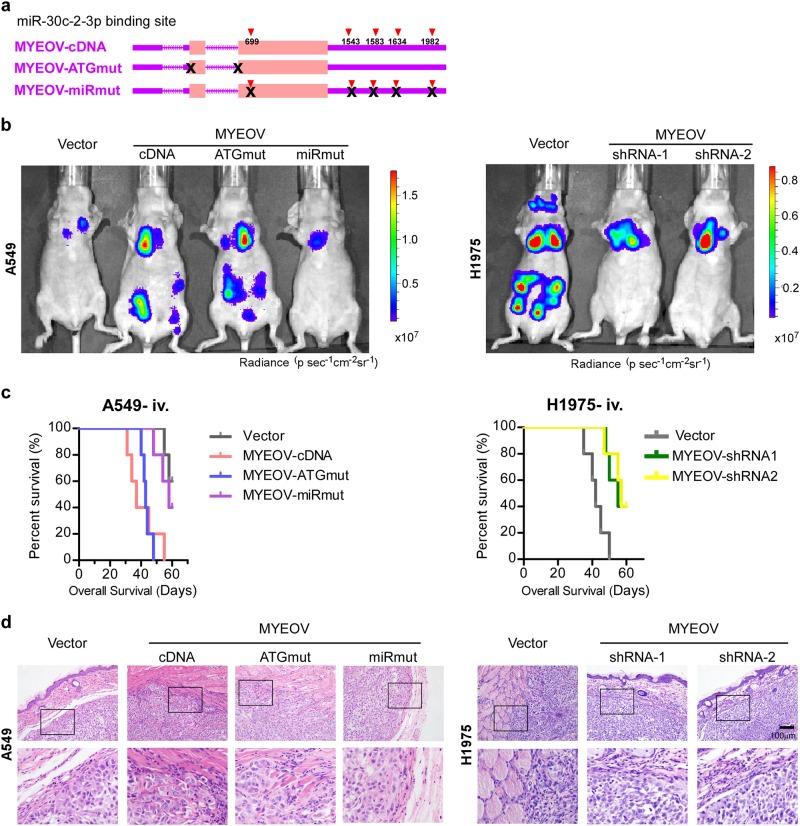
MYEOV transcript enhances invasion and metastasis of NSCLC. **a** Schematic outlining of the MYEOV-cDNA (expression constructs containing the entire MYEOV cDNA transcript), MYEOV-ATGmut (with the start codon mutated), MYEOV-miRmut (MYEOV with mutation of all five predicted miR-30c-2-3p binding sites). **b** Representative bioluminescent images of systemic metastases are shown (5 mice in each group). **c** Overall survival time of mice in indicated groups in the i.v. inoculation model (5 mice in each group). **d** Both the xenografted tumors formed by indicated cells and adjacent subcutaneous tissues were excised (5 mice in each group). Sections were H&E stained to visualize the tumor structure and boundaries. Scale bar: 100 μm

To elucidate whether MYEOV transcript operates as a ceRNA for miR-30c-2-3p in NSCLC progression, in vivo gain-of-function studies were performed by overexpressing wild-type full-length MYEOV cDNA (MYEOV-cDNA), MYEOV cDNA carrying mutation in the in-frame start codon (MYEOV-ATGmut), and MYEOV with mutation of all five predicted miR-30c-2-3p binding sites (MYEOV-miRmut), respectively, in A549, which expresses low-level endogenous MYEOV transcript (Fig. 3a and Supplementary Figure S5a). In an experimental metastasis model, mice intravenously injected with A549-luci-MYEOV-cDNA or A549-luci-MYEOV-ATGmut cells caused metastases in various distal organs at day 28, while mice transplanted with A549-luci-Vector cells or A549-luci-MYEOV-miRmut cells caused only limited pulmonary metastases (Fig. 3b). A549-luci-MYEOV-cDNA or A549-luci-MYEOV-ATGmut cell injected mice started to die at day 31 and day 40, respectively, and none was alive by day 55 (Fig. 3c). Mice injected with A549-luci-MYEOV-miRmut cell or vector control cell started to die at day 48 and day 55, respectively, and some of them still remained alive (2/5 for MYEOV-miRmut and 3/5 for vector control) at the end of the experiments (day 60) (Fig. 3c). In comparison, silencing endogenous MYEOV transcript in H1975 cells, which expressed MYEOV transcript at an elevated level with its DNA copy number amplified, could inhibit the metastatic ability of H1975 cells and increased animal survival (Fig. 3b, c). Subcutaneous xenograft studies showed that A549-Vector or A549-MYEOV-miRmut cells formed well circumscribed tumor with clear edges, while tumors formed by A549-MYEOV-cDNA or A549-MYEOV-ATGmut cells exhibited highly invasive morphology with obscure boundary (Fig. 3d). Meanwhile, silencing MYEOV transcript in H1975 inhibited subcutaneous tumor invasiveness (Fig. 3d).

**Fig. 4:**
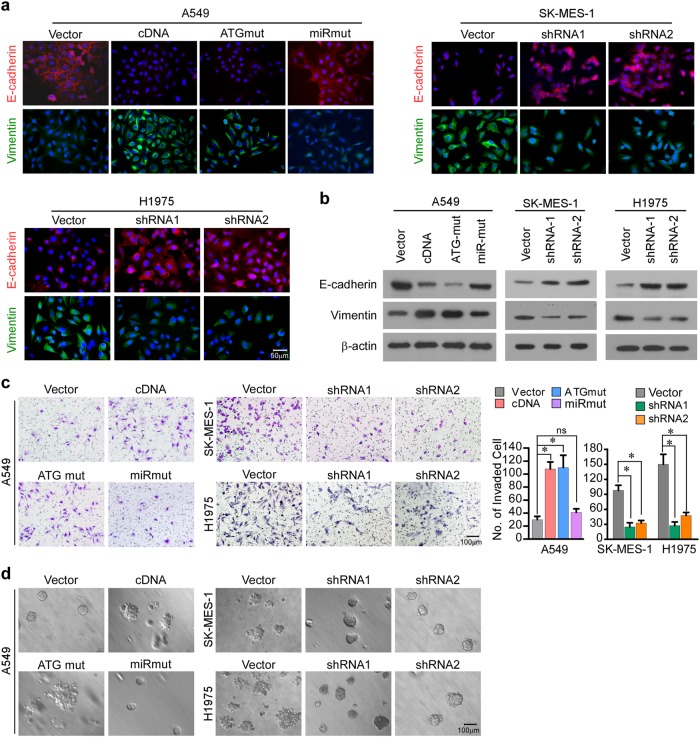
MYEOV transcript promotes NSCLC invasiveness and metastasis in vitro. **a** The protein levels of EMT markers in indicated cells examined by immunofluorescence. Scale bar: 50 μm. **b** The protein levels of EMT markers in indicated cells examined by WB analysis. **c** Invasiveness as measured by the Matrigel Transwell assay. Scale bar: 100 μm. Columns, number of cells invaded across the membrane (each bar represents the mean ± SD derived from three independent experiments, one-way ANOVA followed by Dunnett’s multiple comparison test. *P < 0.05; ns, not significant). **d** Representative micrographs of indicated cells grown on the Matrigel in 3-D spheroid invasion assay. Scale bar: 100 μm

Next, in vitro experiments were conducted and revealed that A549 cells transduced with MYEOV-cDNA or MYEOV-ATGmut exhibited lower E-cadherin and higher Vimentin levels than vector cells and MYEOV-miRmut (Fig. 4a, b). In addition, A549 cells with overexpressed MYEOV-cDNA or MYEOV-ATGmut increased the number of invaded NSCLC cells in Matrigel-coated Transwell assay and exhibit more aggressive phenotype in 3-D spheroid invasion assay, as compared with cell transduced with vector or MYEOV-miRmut (Fig. 4c, d). Meanwhile, silencing MYEOV transcript in SK-MES-1 and H1975 cells markedly weakened EMT and invasive capabilities of the cells (Fig. 4a–d). Collectively, our in vivo and in vitro data reveal that the ceRNA activity of MYEOV transcript plays a pivotal role in promoting the invasion and metastasis of NSCLC.

### MYEOV ceRNA activates TGF-β signaling

**Fig. 5:**
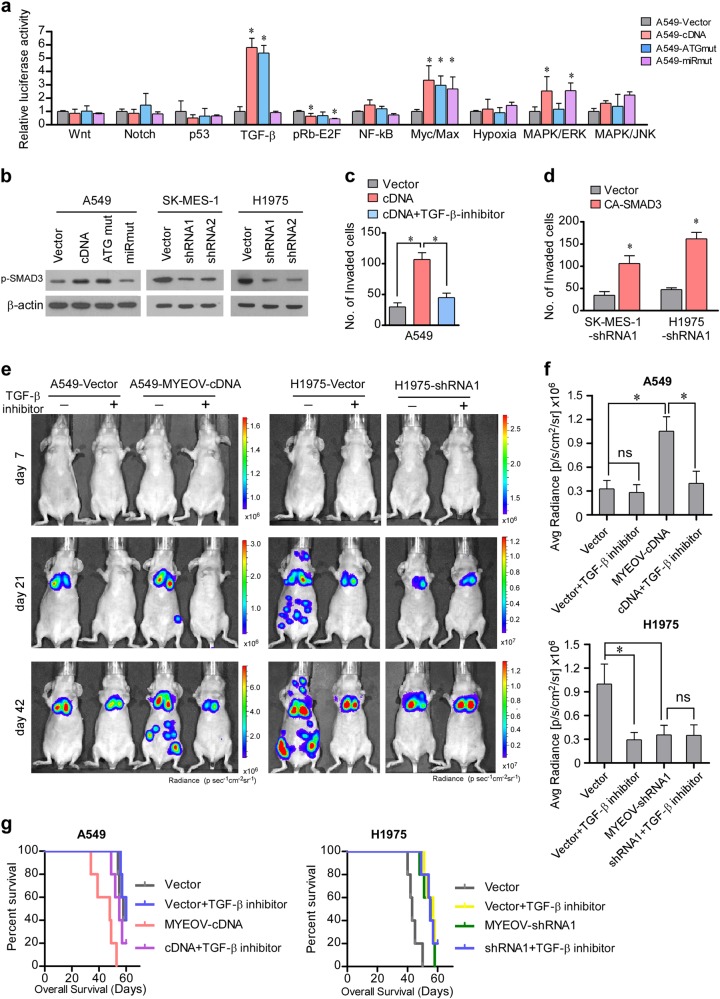
MYEOV activates TGF-β signaling to trigger invasion-metastasis cascade. **a** Cignal 10-Pathway Reporter Array data obtained in A549 cells upon overexpression of MYEOV-cDNA, MYEOV-ATGmut, or MYEOV-miRmut (each bar represents the mean ± SD derived from three independent experiments, two-tailed Student’s *t* test. *P < 0.05). **b** WB analysis of p-SMAD3 in the indicated cells; β-actin was used as the loading control. **c** The number of invaded cells induced by MYEOV transcript following treatment with TGF-β inhibitor (LY2109761) (each bar represents the mean ± SD derived from three independent experiments, one-way ANOVA followed by Bonferroni’s multiple comparison test. *P < 0.05). **d** MYEOV transcript silencing-induced suppression of cell invasion was abrogated by transfection of CA-SMAD3 (each bar represents the mean ± SD derived from three independent experiments, two-tailed Student’s *t* test. *P < 0.05). **e** Representative bioluminescent images of tumor metastases in response to LY2109761 treatment. **f** Quantification of bioluminescent signal (each bar represents the mean ± SD derived from three independent experiments, one-way ANOVA followed by Bonferroni’s multiple comparison test. *P < 0.05). **g** Overall survival time of mice in indicated groups in the i.v. inoculation model (5 mice in each group)

To understand the mechanism underlying the ability of MYEOV transcript to promote invasion and metastasis in NSCLC, Cignal Finder 10 Pathway Reporter Arrays were employed to identify signaling pathways responding to MYEOV regulation. As shown in Fig. 5a, we found that the transactivating activity of TGF-β signaling was potently enhanced by overexpression of MYEOV-cDNA or MYEOV-ATGmut, but not MYEOV-miRmut, suggesting that TGF-β signaling can be modulated by the ceRNA activity of MYEOV transcript. In agreement with the gain-of-function experiments, silencing MYEOV decreased the TGF-β-driven luciferase reporter activity (Supplementary Figure S5b). Furthermore, the expression level of p-SMAD3 was markedly increased in MYEOV-cDNA or MYEOV-ATGmut transcript-overexpressing cells, but decreased when MYEOV transcript was suppressed (Fig. 5b). In addition, the expressions of 4 classical TGF-β target genes (PAI-1, ANGPTL4, MMP9, and CDH1) were specifically correlative with MYEOV transcript expression (Supplementary Figure S5c). Next, we examined the importance of the TGF-β pathway in MYEOV transcript-mediated effects using TGF-β inhibitor and TGF-β constitutively active plasmid. As shown in Fig. 5c, d, the invasiveness of MYEOV transcript-overexpressing cells was attenuated upon treatment with the TGF-β inhibitor LY2109761, which blocks the TGFBR1 and TGFBR2 kinase activity, while constitutively active SMAD3 mutants (CA-SMAD3) [9] reversed the inhibitory effects of MYEOV knockdown on cell invasion. In our in vivo experimental metastasis model, A549 cells with MYEOV transcript overexpressed formed more metastatic nodules and showed a shorter OS time as compared with the vector-control cells, whereas silencing MYEOV in H1975 cells remarkably decreased metastasis and increased animal survival (Fig. 5e–g). Notably, LY2109761 treatment significantly suppressed the metastatic capability of A549-MYEOV-cDNA and H1975-vector cells and prolonged animal survival, but had no obvious effects on A549-vector cells and H1975 cells with MYEOV knocked down (Fig. 5e–g), further suggesting that the TGF-β/SMAD pathway mediates the pro-metastatic effect of MYEOV transcript.

### MYEOV attenuates miRNA-mediated suppression of TGFBR2 and USP15

**Fig. 6:**
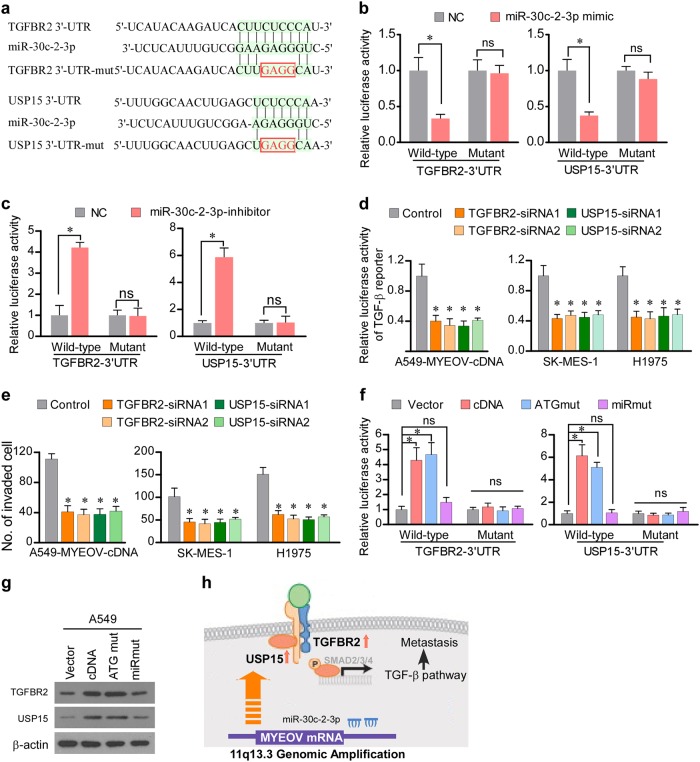
MYEOV attenuates miRNA-mediated suppression of TGFBR2 and USP15. **a** Binding sites of miR-30c-2-3p in TGFBR2-3′-UTR and USP15-3′-UTR. **b** Relative luciferase activities in indicated cells transfected with miR-30c-2-3p mimic and luciferase reporters containing TGFBR2-3’UTR or USP15-3′UTR (each bar represents the mean ± SD derived from three independent experiments, two-tailed Student’s *t* test. *P < 0.05; ns, not significant). **c** The reporters containing TGFBR2-3′-UTR or USP15-3′-UTR were co-transfected with miR-30c-2-3p inhibitor, and luciferase activities were assessed after 48 h (each bar represents the mean ± SD derived from three independent experiments, two-tailed Student’s *t* test. *P < 0.05; ns, not significant). **d** Effects of silencing TGFBR2 or USP15 in indicated cells on luciferase activities of the TGF-β responsive reporter (each bar represents the mean ± SD derived from three independent experiments, one-way ANOVA followed by Dunnett’s multiple comparison test. *P < 0.05). **e** Effects of silencing TGFBR2 or USP15 on cell invasion as measured by Transwell invasion assay in the indicated NSCLC cells (each bar represents the mean ± SD derived from three independent experiments, one-way ANOVA followed by Dunnett’s multiple comparison test. *P < 0.05). **f** Luciferase assay of reporters for pGL3-TGFBR2-3′-UTR or pGL3-USP15-3′-UTR in indicated cells, co-transfected with MYEOV-cDNA, MYEOV-ATGmut or MYEOV-miRmut (each bar represents the mean ± SD derived from three independent experiments, one-way ANOVA followed by Dunnett’s multiple comparison test. *P < 0.05; ns, not significant). **g** WB analysis performed for TGFBR2 and USP15 with β-actin as loading control. **h** Model diagram of proposed MYEOV-mediated regulation of TGF-β signaling in NSCLC

To further elucidate the mechanism via which the ceRNA activity of MYEOV enhances metastasis of NSCLC, TargetScan was used to screen the putative targets of miR-30c-2-3p. Among the targets indicated by the TargetScan analysis, two target genes, i.e., TGFBR2 and deubiquitinating enzyme USP15 (Fig. 6a), which have been previously recognized as components of the TGF-β pathway [33], were further investigated for their possible contribution to MYEOV modulation of TGF-β signaling. To demonstrate that miR-30c-2-3p directly targets TGFBR2 and USP15, we constructed luciferase reporters containing wild type (WT) or mutant (Mut) 3′-UTR of TGFBR2 and USP15 (Fig. 6a). Luciferase assays demonstrated that the activity of luciferase linked to the 3′-UTR of TGFBR2 or USP15 was suppressed by ectopic miR-30c-2-3p, whereas inhibition of miR-30c-2-3p increased the luciferase reporter activities (Fig. 6b, c). Meanwhile, the use of 3’-UTR reporters containing mutations in the miR-30c-2-3p binding sites showed that these mutations abolished the effect of deregulated miR-30c-2-3p on the luciferase reporter (Fig. 6b, c). Immunoblotting analysis consistently revealed that the expression levels of TGFBR2 and USP15 were reduced in miR-30c-2-3p-overexpressing cells, whereas miR-30c-2-3p inhibition upregulated the levels of these proteins (Supplementary Figure S6a). To investigate the functional significance of TGFBR2 and USP15 in the TGF-β signaling and invasive capability of NSCLC cells, the effects of depleting TGFBR2 and USP15 were investigated. As shown in Fig. 6d, e, individually silencing TGFBR2 or USP15 inhibited the transactivating activity of TGF-β signaling and cell invasiveness. Moreover, because MYEOV transcript functions as a ceRNA for miR-30c-2-3p, we hypothesized that it might influence the expression of TGFBR2 and USP15. As shown in Fig. 6f, ectopic expression of MYEOV-cDNA or MYEOV-ATGmut cDNA increased the luciferase activity of the reporter vector containing USP15-3′-UTR or TGFBR2-3′-UTR in A549 cells, whereas cells transduced with MYEOV-miRmut did not cause such changes. Furthermore, restoration of miR-30c-2-3p could reverse the increased luciferase activity of TGFBR2-3′-UTR or USP15-3′-UTR reporter and enhanced invasive capability caused by MYEOV transcript (Supplementary Figures S6b, c). Moreover, the expression levels of TGFBR2 and USP15 were upregulated in cells ectopically expressing MYEOV-cDNA or MYEOV-ATGmut when compared with vector control cells, whereas introduction of mutant miRNAs binding sites of MYEOV attenuated this effect (Fig. 6g). In addition, depletion of MYEOV transcript decreased the expression of TGFBR2 and USP15 (Supplementary Figure S6a). Taken together, these data suggest a pivotal role of MYEOV transcript in modulating TGFBR2 and USP15 by competitively binding miR-30c-2-3p, which activates TGF-β signaling and promotes cell invasion and metastasis (Fig. 6h).

### Clinical relevance of MYEOV with TGF-β signaling in NSCLC

**Fig. 7:**
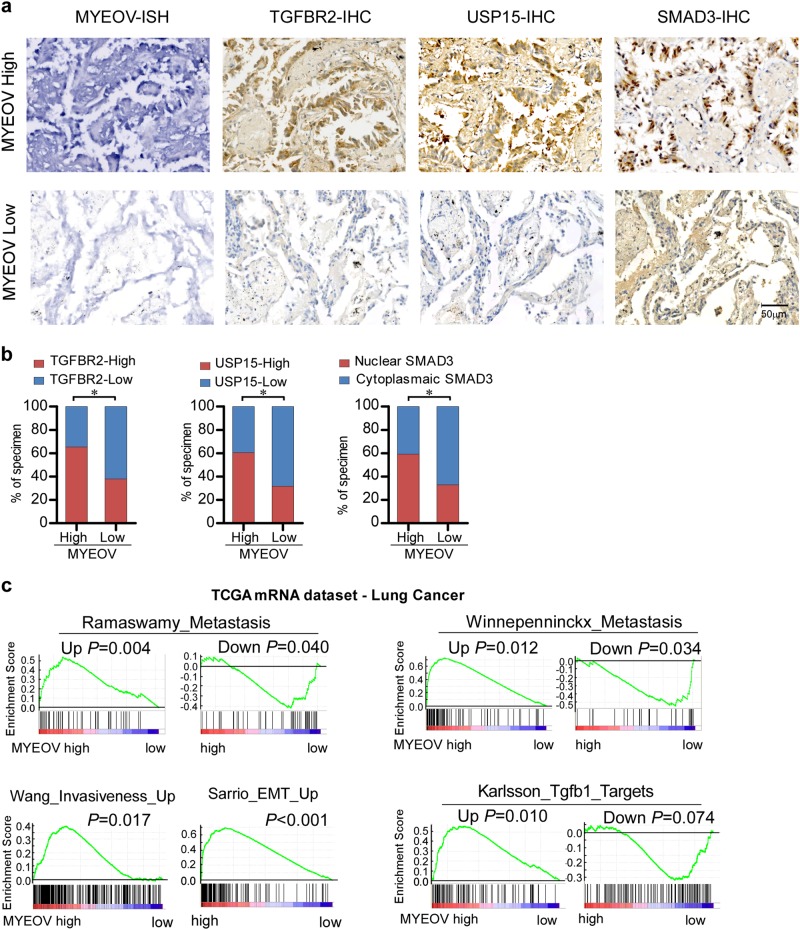
Clinical relevance of MYEOV with expression levels of TGFBR2 and USP15 and activation of TGF-β signaling. **a** Expression of MYEOV is associated with TGFBR2 and USP15 expression levels and localization of SMAD3 in clinical NSCLC specimens. Two representative cases (high and low MYEOV) are shown. **b** Percentage of specimens showing high- or low- MYEOV expression in relation to the expression levels of TGFBR2, USP15 and nuclear or cytoplasmic SMAD3. The χ^2^-test was used to analyze statistical significance. *P < 0.05. **c** GSEA plot showing MYEOV expression in relation to metastasis-associated gene signature and TGF-β-activated gene signature in the TCGA lung cancer dataset

We next examined whether the experimentally observed upregulation of MYEOV transcript and its mediation of TGF-β activation were clinically relevant in NSCLC. IHC assays were performed in 160 cases clinical in NSCLC specimens and results exhibited that the protein expression levels of TGFBR2, USP15, and nuclear-localized SMAD3 were significantly higher in MYEOV transcript-high expression group than those in the MYEOV transcript-low expression group (Fig. 7a, b), indicating that the transcript level of MYEOV significantly correlated with TGFBR2 and USP15 expression as well as the nuclear location of SMAD3 in clinical NSCLC. Additionally, MYEOV transcript expression was correlative with EMT and metastasis geneset signatures in the TCGA NSCLC dataset (Fig. 7c). We also found that MYEOV expression positively correlated with TGF-β target genes. As shown in Fig. 7c, genes that can be upregulated by TGF-β signaling are significantly enriched in MYEOV-high expression group, whereas TGF-β signaling downregulated genes are enriched in MYEOV-low expression group. Taken together, these results suggest a clinical link between overexpression of MYEOV and NSCLC progression.

## Discussion

A key finding of our present study is that the MYEOV transcript could potentiate NSCLC invasion and metastasis through a ceRNA-based regulatory mechanism independent of its protein-coding function. This finding extends our current understanding on the molecular mechanism mediating NSCLC progression, uncovers a new layer of regulatory modality for TGF-β signaling, and identifies a new mode of action for metastasis-associated genes in NSCLC.

The accumulation of genetic changes as a hallmark of NSCLC development usually not only results in increased expression and/or overactivation of oncogenic genes but also represents useful prognostic markers or therapeutic targets [34, 35]. Notably, amplification of chr11q13.3 is frequently found and associated with poor clinical outcome in a wide spectrum of tumor types, including NSCLC [13–16]. Within this frequently present amplicon, we focused on the MYEOV, as it exhibits as the most upregulated transcript in this amplicon. Our data showed that the expression of MYEOV in parallel with 11q13.3 amplification, suggesting that the MYEOV overexpression might be attributable to a genomic gain of the gene in 11q13.3. Remarkably, our data established that MYEOV transcript was overexpressed in NSCLC and its overexpression predicted poor clinical outcome of the patients with the disease. Consistent with its overexpression, we found that MYEOV transcript exerted critical functions in the invasion and metastasis of NSCLC cells. These support the oncogenic role of MYEOV transcript in NSCLC.

Accumulating evidence has demonstrated that coding and noncoding RNAs can act as ceRNAs to sequester miRNAs, thereby liberating their mRNA targets from suppression [30, 36–38]. Here, we identify that MYEOV transcript exhibits decoy activity for miR-30c-2-3p, and by doing so, upregulates the expression of its targets, leading to the aggressive phenotype of NSCLC. Several lines evidence collected in our current study support the ceRNA activity of MYEOV transcript. We searched potential miRNA-recognition elements in the MYEOV transcript sequence and found 5 putative binding sites for miR-30c-2-3p, and RIP and luciferase assays demonstrated that miR-30c-2-3p is indeed a direct binding partner to MYEOV transcript. More interestingly, the biological effects of the MYEOV transcript were largely miR-30c-2-3p-dependent, as MYEOV cDNA and MYEOV cDNA carrying mutation in the in-frame start codon promoted invasion and metastasis of NSCLC, but not MYEOV with mutation of all five predicted miR-30c-2-3p binding sites. In addition, overexpression of MYEOV transcript increased the levels of targets of miR-30c-2-3p, namely, TGFBR2 and USP15, while its depletion produced a decrease in the expression of both of them. These data allowed us to conclude that MYEOV transcript is indeed a decoy for miR-30c-2-3p in NSCLC. Furthermore, as we also observed that the expression of MYEOV is upregulated in other cancer types, MYEOV may also function as a ceRNA in these malignancies.

Indeed, it is well recognized that one specific miRNA can regulate a cohort of target genes involved in different signaling pathways [39, 40]. While it is possible that MYEOV exerts its function via more than one pathway regulated by miR-30c-2-3p, the fact that TGF-β inhibitor significantly abrogated MYEOV-induced metastasis, supports the notion that MYEOV functions as a ceRNA to regulate TGF-β signaling. Whether the oncogenic role of MYEOV also requires additional mechanisms remains to be clarified in future studies.

The aberrant activation of TGF-β signaling has been demonstrated in tumors tissues and cancer cell lines with metastatic phenotype in NSCLC [9, 10]. However, unlike in other cancer types such as colon cancer, genetic mutations of its component genes, such as TGFBR2 and SMAD2/4, are rare [11, 12]. Of note, TGFBR2 and USP15 have been identified as mediating molecules that play important roles in the activation of TGF-β signaling [8, 33]. USP15, a deubiquitinating enzyme, plays an important regulatory role in the TGF-β signaling. USP15 can form complex with SMAD7 and SMAD specific E3 ubiquitin protein ligase 2 (SMURF2), which subsequently deubiquitinates and stabilizes TGFBR1 and consequently causes activation of TGF-β signaling [33]. Our mechanistic study revealed that dysregulated expression of TGFBR2 and USP15 is coordinately and simultaneously modulated by ectopic expression of MYEOV transcript, which absorbs, and thereby inhibits the free activity of, miR-30c-2-3p, leading to TGF-β-induced aggressive phenotype of NSCLC cells. Such a significance of MYEOV transcript in NSCLC metastasis was demonstrated in both in vitro as well as in vivo experiments. Tumor-bearing mice that exhibit high MYEOV expression revealed shorter survival time. In in vivo experimental metastasis model, cells with overexpression of MYEOV transcript formed more metastatic nodules and were sensitive to TGF-β inhibitor therapy. Whereas, metastatic nodules formed by cells with MYEOV depletion showed only limited response to treatment of TGF-β inhibitor. Thus, our study proposes that MYEOV may also represent a potential predictor for TGF-β inhibitory therapy against NSCLC. Future preclinical studies and prospective clinical trials will be needed to address whether MYEOV level can be used to predict the benefit obtainable from TGF-β inhibitor therapies in NSCLC.

## Materials and methods

### Cell culture

Lung cancer cell lines, including A549, Calu-1, Calu-3, NCI-H292, SK-MES-1, NCI-H460, NCI-H596, NCI-H358, NCI-H1650, NCI-H1299, NCI-H1975, and NCI-H2009, were obtained from the American Type Culture Collection (ATCC; Manassas, VA), and maintained in DMEM medium (Invitrogen, Carlsbad, CA) supplemented with 10% fetal bovine serum (Corning, Corning, NY). Primary normal lung epithelial cells (NLEC) were obtained according to a previous report [41]. The BEAS-2B immortalized human bronchial epithelial cell line (Shanghai Institutes of Biological Sciences, Shanghai, China) was cultured in KSFM medium (Invitrogen) as instructed by the provider. All cell lines were authenticated by short tandem repeat (STR) fingerprinting at Medicine Laboratory of Forensic Medicine Department of Sun Yat-Sen University (Guangzhou, China) and were verified to be mycoplasma-free.

### Clinical specimens and patient information

Paraffin-embedded human NSCLC specimens used in this study were histopathologically diagnosed at the Sun Yat-sen University Cancer Center (SYSUCC). Clinical NSCLC specimens and the paired adjacent noncancerous lung specimens were frozen and stored in liquid nitrogen until further use. Adjacent non-tumor specimens were obtained from a standard distance (3 cm) from resected neoplastic tissues of NSCLC patients who underwent surgical lung resection and confirmed by pathological evaluation. For the use of these clinical materials for research purposes, prior patients’ consents and approval from the Institutional Research Ethics Committee were acquired. Clinical information of a cohort of 160 cases of NSCLC specimens are presented in Supplementary Table S3.

### RNA extraction and qRT-PCR

Total RNA extraction from NSCLC cells and tissues was performed using TRIzol reagent (Invitrogen, Carlsbad, CA). For mRNA quantification, first-strand cDNA was generated by MMLV transcriptase (Promega, Madison, WI) using random primers. The expression of mRNA was assessed by qRT-PCR with iTaq Universal SYBR Green Supermix (Bio-Rad, Hercules, CA) and CFX96 real-time PCR detection system (Bio-Rad). The relative expression levels of mRNA were normalized to GAPDH expression, and fold changes in expression were calculated using the 2^−ΔΔCt^ method. Primers for each transcript were commercially synthesized and provided by Invitrogen (Shanghai, China), whose sequences are listed in Supplementary Table S4. For miRNA analysis, specific primers for each mature miRNA were purchased from RiboBio (Guangzhou, China) and used according to the manufacturer’s instructions. For exact quantification of gene copy numbers per cell, MYEOV expressing vector and miDETECTTM miR-30c-2-3p standard RNA synthesized by RiboBio (Guangzhou, China) were used as standard templates to formulate standard curves with a limiting dilution approach, and then the exact copy numbers of MYEOV and miR-30c-2-3p per cell were calculated based on their molecular weights and cell counts.

### Plasmids, infection, and transfection

Plasmids were constructed using standard methods. MYEOV-cDNA and MYEOV-ORF were amplified from the cDNA preparation of H1975 cells using Pfu polymerase (Agilent Technologies, Santa Clara, CA) and cloned into the pSIN-EF2 lentivirus plasmid. The MYEOV-5’-UTR-mutation, MYEOV-ATG-mutation, MYEOV-miR-mutation were generated using the QuikChange II Site Directed Mutagenesis Kit (Agilent). The 3′-UTRs of TGFBR2 and the USP15 genes containing the predicted potential miRNA binding sites were amplified and ligated downstream of the luciferase gene in a pGL3 control vector (Promega); the mutant 3′-UTRs of TGFBR2 and the USP15 carrying mutated sequences in the complementary sites in the seed region for miR-30c-2-3p were synthesized by Genewiz (Suzhou, China) and were cloned into the pGL3 control vector (Promega). The shRNA sequence of MYEOV was ligated in the pSUPER-retro-puro. pMS2-GFP and pSL-MS2-12× were obtained from Addgene (Cambridge, MA). pSL-MYEOV-cDNA-MS2-12× was constructed by inserting MYEOV-cDNA into pSL-MS2-12×. A constitutively active SMAD3 mutant, namely, the CA-SMAD3 (mutation of three C-terminal serine residues in SMAD3 to aspartic acid) construct, was subcloned into the pcDNA3.1 vector plasmids (Invitrogen). Recombinant retrovirus production and infection were performed as previously described [42]. Following transduction, puromycin (1.5 μg mL^−1^) was used as a selection antibiotic to select the infected cells for 7 days. Transfection of plasmids or oligonucleotides was performed using the Lipofectamine 3000 reagent (Invitrogen) by following the manufacturer’s instruction. For detailed description of primers for plasmid construction and sequences of synthetic oligonucleotides, see Supplementary Table S5 and Table S6.

### 5′ and 3′ rapid amplification of cDNA ends (RACE)

5′-RACE and 3′-RACE analyses were employed to determine the transcriptional initiation and termination sites of MYEOV using a SMARTer™ RACE cDNA Amplification Kit (Clontech, Palo Alto, CA) according to the manufacturer’s instructions. The PCR gene-specific primers used for the RACE analysis are summarized in Supplementary Table S7.

### Western blotting (WB) and immunofluorescence assays

WB and immunofluorescence assays were performed, respectively, according to corresponding standard methods [43]. The antibodies used in the study were as follows: anti-MYEOV (Sigma, HPA012949), anti-SMAD3 (Cell Signaling Technology, #9523), anti-p-SMAD3 (Abcam, ab52903), anti-USP15 (Cell Signaling Technology, #66310), anti-TGFBR2 (Abcam, ab61213), anti-E-cadherin (Abcam, ab1416), anti-Vimentin (BD, #550513), anti-β-actin (Cell Signaling Technology, #4970). Fluorescence images were captured using the Axio Imager A1 microscopy system (Carl Zeiss, Oberkochen, Germany).

### RNA immunoprecipitation (RIP)

RIP was performed as previously described [44]. Cells were transfected with pMS2-GFP and pSL-MYEOV-cDNA-MS2-12× or pSL-MS2-12×, harvested for RIP by using a GFP antibody (Abcam, Cambridge, MA) and the Magna RIP RNA-Binding Protein Immunoprecipitation Kit (Millipore, Bedford, MA).

### Cignal finder cancer 10-pathway reporter array

The Cignal Finder Cancer 10-Pathway Reporter Array (Qiagen, Dusseldorf, Germany) was performed to analyze the effects of MYEOV on various key signaling pathways involved in cancer development and progression. Briefly, indicated cells were transfected with transcription factor-responsive reporter constructs according to the manufacturer’s instruction. After 48 h of transfection, the luciferase assay was performed using the Dual-Luciferase Reporter Assay System (Promega) following the instruction provided by the manufacturer.

### Animal experiments

All experimental procedures were approved by the Institutional Animal Care and Use Committee of Sun Yat-sen University. For the experimental metastasis model, the indicated cells (2 × 10^6^) labeled with firefly luciferase were intravenously (i.v.) injected into the tail veins of nude mice. TGF-β inhibitor, LY2109761 (Selleck Chemicals, Houston, TX), was administered orally at 50 mg kg^−1^ twice daily for five consecutive days followed by a 2-day rest (5 days on/2 days off) every week until the end of observation. Metastasis progression was monitored and quantified using a noninvasive bioluminescence Xenogen IVIS Spectrum System (Caliper Life Sciences, Boston, MA, USA). For establishment of subcutaneous invasion model, 5 × 10^6^ cells of indicated cells were subcutaneously implanted into 7-week-old BALB/c nude mice. Tumor formation in nude mice was monitored over a 4-week period, and then the mice were sacrificed for tumor excision and subjected to pathological examination. At least five nude mice per group were used to ensure the adequate power and each mouse with different weight was randomly allocated. Bioluminescent imaging of primary tumors and metastases was not performed in a blinded manner.

### Fluorescence in situ hybridization (FISH)

Dual-color FISH assays were conducted using following probes: MYEOV/CEP11 probe mixture containing homebrewed MYEOV DNA labeled with SpectrumOrange; The chromosome 11 control probe CEP11 (centromere enumeration) labeled with SpectrumGreen. Whole-tissue sections were deparaffinized, boiled in pretreatment buffer (70% formamide, 2× saline-sodium citrate buffer) at 100 °C for 15 min, digested with proteinase K for 10 min. The FISH probe was applied and slides were sealed with rubber cement. Following a denaturation step, slides were incubated overnight at 37 °C. Slides were washed in wash Buffer (2× SSC, 0.3% NP40, pH 7-7.5) and counterstained with 1000 ng mL^−1^ 4′,6-diamidino-2-phenylindole (DAPI) (Sigma). Signals from MYEOV compared to those from chromosome 11 were analyzed in at least ten cells. The mean MYEOV, CEP11 gene copy number per nucleus and the MYEOV/CEP11 ratio were reported. FISH MYEOV gene amplification in this study was defined as both MYEOV/CEP11 ratio ≥ 2 and MYEOV gene copy number ≥ 4. Both criteria were required to be met to rule out samples with MYEOV/CEP11 ratio ≥ 2 merely due to isolation loss of CEP11.

### Immunohistochemical analysis (IHC)

IHC assay was performed and quantitatively analyzed as previously described [45, 46]. Paraffin-embedded NSCLC serial sections were analyzed with antibodies against TGFBR2 (Abcam, ab61213), USP15 (Abcam, ab71713) and SMAD3 (Abcam, ab28379), respectively. The degree of immunostaining of target proteins was evaluated and scored based on the proportions of tumor cells stained positively and the intensity of the staining by two independent observers.

### Bioinformatics analysis

MiRNA target prediction was performed using Targetscan (http://targetscan.org/vert_60/). Gene set enrichment analysis (GSEA) was performed by the GSEA 2.0.9 software using methods described in previous reports [47]. All TCGA datasets (lluminaHiSeq_RNASeqV2 data and DNA copy-number using Affymetrix Genome-Wide Human SNP 6.0 array data) were downloaded from the TCGA data portal (https://cancergenome.nih.gov/). Clinical information regarding the enrolled patient specimens is presented in Supplementary Table S8. CNVs analysis was performed using the Genomic Identification of Significant Targets in Cancer (GISTIC2.0) program [48]. Microarray data (GSE5843 and GSE50081) were downloaded from the Gene Expression Omnibus (GEO) database (http://www.ncbi.nlm.nih.gov/geo/).

### Statistical analysis

All statistical analyses, except for microarray data, were performed using the SPSS 19.0 (IBM) statistical software package. Survival curves were analyzed by the Kaplan–Meier plot with a log rank test to assess significance (cutoff by median expression levels of MYEOV). Univariate and multivariable survival analyses were performed using the Cox regression method. Comparisons between two groups were performed using the Student’s *t* test. For pairwise multiple comparisons, one-way ANOVA followed by Dunnett’s multiple comparison test or Bonferroni’s post-hoc test was used as appropriate. Sample size was determined by power analysis to achieve a minimum effect size of 0.5 with *P* < 0.05 and all sample sizes were appropriate for assumption of normal distribution. Variance within each group of data was estimated and was similar between compared groups. Data analysis was performed by two independent investigators who were blinded to the sample groups. In all cases, *P* < 0.05 was considered statistically significant.

## Electronic supplementary material

Supplementary Materials and Methods

Supplementary Figure Legends

Supplementary Table

Supplementary Figure 1

Supplementary Figure 2

Supplementary Figure 3

Supplementary Figure 4

Supplementary Figure 5

Supplementary Figure 6
