# Supplementary material for: MYEOV functions as an amplified competing endogenous RNA in promoting metastasis by activating TGF-β pathway in NSCLC
Source: Oncogene. 2018 Sep 4;38(6):896–912. doi: 10.1038/s41388-018-0484-9 (PMC6756124; doi:10.1038/s41388-018-0484-9)
Supplement: Supplementary file 1 — Supplementary Materials and Methods [file 41388_2018_484_MOESM1_ESM.docx]

**Supplementary Materials and Methods**

**Transwell matrix penetration assay**

Cells (2×10^4^) were plated on the top side of polycarbonate Transwell coated with Matrigel (10%, BD, Franklin Lakes, NJ) and incubated at 37°C for 48 h, followed by removal of cells inside the upper chamber with cotton swabs. Invasive cells on the lower membrane surface were fixed in 4% paraformaldehyde, stained with hematoxylin, and counted (Ten random 200× fields per well). Cell counts are expressed as the mean number of cells per field of view.

**Luciferase reporter assay**

Cells were seeded in triplicates in 24-well plates and allowed to settle for 24 h. Indicated plasmids plus 1ng pRL-TK Renilla plasmid was transfected into the cells using the Lipofectamine 3000 reagent (Invitrogen) according to the manufacturer’s recommendation. Forty-eight hours after transfection, dual-luciferase reporter assay was performed according to the manufacturer’s instruction (Promega).

**Three-dimension (3-D) spheroid invasion assay**

Cells (1×10^4^) were mixed with 20% Matrigel (BD) and seeded in 24-well plates coated with 100% Matrigel (BD), and medium was changed every other day. Pictures were taken under microscope at various time points.

**LNA-based *in situ* hybridization (ISH)**

LNA ISH was performed using the miRCURY LNA miRNA ISH Optimization Kit (Exiqon, Vedbaek, Denmark) according to the protocol provided by the manufacturer. Briefly, the sections were deparaffinized and then deproteinated by incubation with 15 mg ml^-1^ proteinase K (Exiqon) for 20 min at 37°C. The endogenous peroxidases were inactivated in 1% H_2_O_2_ for 30 min, and sections were pre-hybridized at 62°C for 30 min in formamide-free Exiqon ISH buffer (Exiqon) and hybridized with DIG-labelled LNA probes for MYEOV (5′-TAATATGGCTGGTGAAAGCACT-3′, 50nM, Exiqon) at 62°C overnight. Slides were then stringently washed, incubated with alkaline phosphatase-conjugated anti-DIG reagent (Roche) for 60 min and then detected by NBT/BCIP reagent. Assessment of the staining was based on the staining intensity and the percentage of positively stained cells by two independent observers and visualized with the IX51 Olympus microscope (Olympus, Center Valley, PA) accompanied with the CellSens image software (Olympus).
