# Supplementary material for: MYEOV functions as an amplified competing endogenous RNA in promoting metastasis by activating TGF-β pathway in NSCLC
Source: Oncogene. 2018 Sep 4;38(6):896–912. doi: 10.1038/s41388-018-0484-9 (PMC6756124; doi:10.1038/s41388-018-0484-9)
Supplement: Supplementary file 2 — Supplementary Figure Legends [file 41388_2018_484_MOESM2_ESM.docx]

**Supplementary Figure Legends**

**Supplementary Figure S1. MYEOV amplification occurs frequently in NSCLC.**

**(a)** mRNA expression of indicated genes in NSCLC tumors and adjacent noncancerous tissue specimens within 3q26.33, 11q13.3 and 8p11.23 amplicons is shown as a histogram. **(b)** The frequency of chr11q13.3 and MYEOV amplification in three independent NSCLC cohorts as indicated. **(c)** Statistical analysis of MYEOV expression in NSCLC versus corresponding adjacent noncancerous tissue in the SYSUCC cohort (two-tailed paired Student’s *t* test. **P*<0.05). **(d)** MYEOV mRNA levels were evaluated in NSCLC tissue with and without 11q13.3 amplification in the SYSUCC cohort. Box plot represents lower quartile; median and upper quartile and whiskers represent 95% confidence interval of the mean (two-tailed Student’s *t* test. **P*<0.05). **(e)** MYEOV DNA copy number and MYEOV mRNA expression level in NSCLC cell lines and relationship between the CNVs of MYEOV and its mRNA expression level (each bar represents the mean ± SD derived from three independent experiments, two-tailed Student’s *t* test. **P*<0.05).

**Supplementary Figure S2. MYEOV amplification and transcript upregulation in various cancer types.**

**(a)** Cross-cancer type copy numbers of MYEOV obtained from TCGA. ESCA: esophageal carcinoma; HNSC: head and neck squamous cell carcinoma; OV: ovarian serous cystadenocarcinoma; BRCA: breast invasive carcinoma; BLCA: bladder urothelial carcinoma; STAD: gastric adenocarcinoma. **(b)** MYEOV mRNA levels were evaluated in tumor samples with and without 11q13.3 amplification in TCGA cross-cancer datasets. Box plot represents lower quartile; median and upper quartile and whiskers represent 95% confidence interval of the mean (one-way ANOVA followed by Bonferroni's multiple comparison test. **P*<0.05). **(c)** The expression levels of MYEOV in adjacent noncancerous tissue and tumor tissue, obtained and analyzed from the TCGA dataset (median with interquartile range, two-tailed unpaired Student’s *t* test. **P*<0.05). **Supplementary Figure S3. 5'-RACE and 3'-RACE of MYEOV transcript.**

**(a)** Electrophoretic gel imaging of PCR products derived from the 5'-RACE and 3'-RACE experiments. The main PCR product is marked by red arrows. **(b)** The representative sequencing of PCR products derived from the 5'-RACE and 3'-RACE experiments. **(c)** Sequence of full-length of MYEOV.

**Supplementary Figure S4. Association between MYEOV transcript and predicted binding miRNAs.**

**(a)** Schematic outlining of predicted binding sites of miR-149-3p, miR-765, and miR-3614-5p in the MYEOV transcript. **(b)** MS2-RIP followed by miRNA qRT-PCR was performed to identify an association between MYEOV transcript and miR-149-3p, miR-765, and miR-3614-5p (each bar represents the mean ± SD derived from three independent experiments, two-tailed Student’s *t* test. **P*<0.05).

**Supplementary Figure S5. MYEOV transcript activates TGF-β signaling.**

**(a)** Expression of MYEOV-cDNA, MYEOV-ATGmut, and MYEOV-miRmut in indicated cells was determined by qRT-PCR. Expression levels are normalized to GAPDH (each bar represents the mean ± SD derived from three independent experiments, one-way ANOVA followed by Dunnett's multiple comparison test. **P*<0.05). **(b)** Relative luciferase activity of TGF-β responsive reporter in SK-MES-1 and H1975 cells with MYEOV depleted. Data presented as the relative ratio of firefly luciferase activity to Renilla luciferase activity (each bar represents the mean ± SD derived from three independent experiments, two-tailed Student’s *t* test. **P*<0.05). **(c)** Relative expression levels of 4 downstream target genes known to be regulated by TGF-β signaling, including PAI-1, ANGPTL-4, MMP-9 and CDH1, in indicated cells, as determined by qRT-PCR. Expression levels are normalized to GAPDH (each bar represents the mean ± SD derived from three independent experiments, two-tailed Student’s *t* test. **P*<0.05).

**Supplementary Figure S6. MYEOV transcript modulates TGFBR2 and USP15 levels.**

**(a)** WB analysis performed for TGFBR2 and USP15, respectively, in indicated cells, with β-actin used as a loading control. **(b)** Relative luciferase activity of luciferase reporters containing TGFBR2-3’UTR or USP15-3’UTR (each bar represents the mean ± SD derived from three independent experiments, one-way ANOVA followed by Bonferroni's multiple comparison test.  **P*<0.05; ns, not significant). **(c)** Quantification of invaded cells using Transwell invasion assay (each bar represents the mean ± SD derived from three independent experiments, one-way ANOVA followed by Bonferroni's multiple comparison test. **P*<0.05).
