# Supplementary material for: MYEOV functions as an amplified competing endogenous RNA in promoting metastasis by activating TGF-β pathway in NSCLC
Source: Oncogene. 2018 Sep 4;38(6):896–912. doi: 10.1038/s41388-018-0484-9 (PMC6756124; doi:10.1038/s41388-018-0484-9)
Supplement: Supplementary file 3 — Supplementary Table [file 41388_2018_484_MOESM3_ESM.docx]

**Supplementary Table S1. Correlation between the clinicopathological features and MYEOV CNV status in SYSCUU cohort**

| **Patient Characteristics** | | **MYEOV-CNV status** | | ***P*-value** |
| --- | --- | --- | --- | --- |
|  |  | **non- Amp** | **Amp** |  |
| **Age** | <65 | 35 | 21 | 0.288 |
|  | ≥65 | 13 | 4 |  |
| **Gender** | male | 35 | 18 | 0.934 |
|  | female | 13 | 7 |  |
| **Clinical Stage** | I | 23 | 1 | 0.002 |
|  | II | 6 | 4 |  |
|  | III | 12 | 11 |  |
|  | IV | 7 | 9 |  |
| **Tumor Size** | T1 | 7 | 1 | 0.237 |
|  | T2 | 23 | 14 |  |
|  | T3 | 14 | 5 |  |
|  | T4 | 4 | 5 |  |
| **Nodes Pathologic** | N0 | 28 | 8 | 0.032 |
|  | N1 | 13 | 7 |  |
|  | N2-3 | 7 | 10 |  |
| **Metastasis Pathologic** | M0 | 41 | 16 | 0.036 |
|  | M1 | 7 | 9 |  |

**Supplementary Table S2. Univariate and multivariate analysis of different prognostic parameters in patients with NSCLC by Cox-regression analysis in TCGA**

| **Patient Characteristics** | **Univariate analysis** | |  | **Multivariate analysis** | |
| --- | --- | --- | --- | --- | --- |
|  | **HR (95%CI)** | ***P*-value** |  | **HR (95%CI)** | ***P*-value** |
| **Age(y)** |  |  |  |  |  |
| <65 | 1.000 | - |  | 1.000 | - |
| ≥65 | 1.335 (0.977-1.824) | 0.070 |  | 1.553 (1.131-2.133) | 0.007 |
| **Clinical Staging** |  |  |  |  |  |
| Stage I | 1.000 | - |  | 1.000 | - |
| Stage II | 1.536 (1.083-2.177) | 0.016 |  | 1.650 (1.161-2.345) | 0.005 |
| Stage III | 2.334 (1.634-3.334) | <0.001 |  | 2.620 (1.824-3.763) | <0.001 |
| Stage IV | 1.826 (0.912-3.657) | 0.089 |  | 2.067 (1.027-4.163) | 0.042 |
| **MYEOV Expression** | |  |  |  |  |
| MYEOV-Low | 1.000 | - |  | 1.000 | - |
| MYEOV-High | 1.528 (1.147-2.035) | 0.004 |  | 1.618 (1.213-2.159) | 0.001 |
| **TGFBR2 Expression** | | |  |  |  |
| TGFBR2-Low | 1.000 | - |  |  |  |
| TGFBR2-High | 1.146 (0.861-1.525) | 0.350 |  |  |  |
| **USP15 Expression** | | |  |  |  |
| TGFBR2-Low | 1.000 | - |  |  |  |
| TGFBR2-High | 1.064 (0.797-1.419) | 0.675 |  |  |  |

**Supplementary Table S3. Clinicopathologic characteristics of studied patients in NSCLC from the SYSUCC cohort**

| **Patient Characteristics** | **cases** | **Percentage** |
| --- | --- | --- |
| **Age** |  |  |
| <65 | 110 | 68.8% |
| ≥65 | 50 | 31.3% |
| **Gender** |  |  |
| male | 116 | 72.5% |
| female | 44 | 27.5% |
| **Clinical Stage** |  |  |
| I | 33 | 20.6% |
| II | 46 | 28.8% |
| III | 65 | 40.6% |
| IV | 16 | 10.0% |
| **T classification** |  |  |
| T1 | 14 | 8.8% |
| T2 | 82 | 51.3% |
| T3 | 53 | 33.1% |
| T4 | 11 | 6.9% |
| **N classification** |  |  |
| N0 | 64 | 40.0% |
| N1 | 47 | 29.4% |
| N2 | 47 | 29.4% |
| N3 | 2 | 1.3% |
| **M classification** |  |  |
| M0 | 144 | 90.0% |
| M1 | 16 | 10.0% |

**Supplementary Table S4. Primers used for qRT-PCR**

|  | **Forward Primer** | **Reverse Primer** |
| --- | --- | --- |
| MYEOV | CTCCAAAGCCGGCAGATCC | TTGTTTCTCTCCCGGTCACC |
| SERPINE1(PAI-1) | TGGTGCTGATCTCATCCTTG | AGAAACCCAGCAGCAGATTC |
| ANGPTL4 | TAGTCCACTCTGCCTCTCCC | GAGATGGCCCAGCCAGTT |
| MMP9 | TTGGTCCACCTGGTTCAACT | ACGACGTCTTCCAGTACCGA |
| CDH1 | GACCGGTGCAATCTTCAAA | TTGACGCCGAGAGCTACAC |
| GADPH | TTGAGGTCAATGAAGGGGTC | GAAGGTGAAGGTCGGAGTCA |

**Supplementary Table S5. Primers used for clone construction**

| **Primers** | **Sequence** |
| --- | --- |
| MYEOV-cDNA-Up | CTAGACTAGTCTTGCCTTGATTCTCCTGTTACTGT |
| MYEOV-cDNA-Down | CGCGGATCCTGAGAGCCAGAACGGAAGTC |
| MYEOV-ORF- Up | CTAGACTAGTATGGCCCTCAGAATCTGC |
| MYEOV- ORF - Down | CGCGGATCCTTCAACAAGTGAGGATGATG |
| MYEOV- shRNA-1 Up | GATCCCCGGCTCTCCATGGAAATTATTTCAAGAGAATAATTTCCATGGAGAGCCTTTTTA |
| MYEOV -shRNA-1 Down | AGCTTAAAAAGGCTCTCCATGGAAATTATTCTCTTGAAATAATTTCCATGGAGAGCCGGG |
| MYEOV- shRNA-2 Up | GATCCCCGAGCTGCCCTGACTATGAATTCAAGAGATTCATAGTCAGGGCAGCTC TTTTTA |
| MYEOV-shRNA-2 Down | AGCTTAAAAAGAGCTGCCCTGACTATGAATCTCTTGAATTCATAGTCAGGGCAGCTCGGG |
| MYEOV-cDNA-Luci- Up | ACTCCGCGGCTTGCCTTGATTCTCCTGTT |
| MYEOV- cDNA-Luci- Down | TCAGCTGCAGTGAGAGCCAGAACGGAAGTC |
| TGFBR2-3’UTR -Luci- Up | ACTCCGCGGAGATGCCTCACTCTGGT |
| TGFBR2- 3’UTR -Luci- Down | TCAGCTGCAGTGTCTTCCTGGCTTGTT |
| USP15-3’UTR-Luci- Up | ACTCCGCGGAAATCATGATACAAGAAACAACCTT |
| USP15- 3’UTR-Luci- Down | TCAGCTGCAGCTCTGTCATGCAGGAGGTTAGTTT |

**Supplementary Table S6. Oligonucleotides used for siRNAs**

| **Gene** | **Oligonucleotide (sense)** |
| --- | --- |
| USP15-siRNA1 | CCTTGGAAGTTTACTTAGTTA |
| USP15-siRNA2 | CCCATTGATAACTCTGGACTT |
| TGFBR2-siRNA1 | CGTTCAGAAGTCGGTTAATAA |
| TGFBR2-siRNA2 | GAAGAATATAACACCAGCAAT |

**Supplementary Table S7. Primers used for 5’RACE and 3’RACE**

| **Primers** | **Sequence** |
| --- | --- |
| MYEOV- 5’RACE | 5'-GTTCAGCTGCTGGGTGACTCGGG-3' |
| MYEOV-3’RACE | 5'-GGGAAGCGTGTTGCAATCCATGAG-3' |

**Supplementary Table S8. Clinicopathologic characteristics of patient samples in TCGA**

| **Patient Characteristics** | **cases** | **Percentage** |
| --- | --- | --- |
| **Age (years)** |  |  |
| >65 | 349 | 38.2% |
| ≥65 | 564 | 61.8% |
| **Gender** |  |  |
| Male | 553 | 60.6% |
| Female | 360 | 39.4% |
| **Histology** |  |  |
| Lung Adenocarcinoma | 453 | 49.6% |
| Lung Squamous Cell Carcinoma | 460 | 50.4% |
| **Tobacco Smoking History** | |  |
| Reformed Smoker | 590 | 64.6% |
| Current Smoker | 223 | 24.4% |
| Lifelong Non-smoker | 80 | 8.8% |
| Unknown | 20 | 2.2% |
| **Clinical Stage** | |  |
| Stage I | 470 | 51.5% |
| Stage II | 252 | 27.6% |
| Stage III | 157 | 17.2% |
| Stage IV | 30 | 3.3% |
| Unknown | 4 | 0.4% |
| **Tumor Size** | | |
| T1 | 253 | 27.7% |
| T2 | 519 | 56.8% |
| T3 | 103 | 11.3% |
| T4 | 36 | 3.9% |
| Unknown | 2 | 0.2% |
| **Nodes Pathologic** |  |  |
| N0 | 580 | 63.5% |
| N1 | 208 | 22.8% |
| N2 | 103 | 11.3% |
| N3 | 7 | 0.8% |
| Unknown | 15 | 1.6% |
| **Metastasis Pathologic** |  |  |
| M0 | 689 | 75.5% |
| M1 | 29 | 3.2% |
| Unknown | 195 | 21.3% |
