# Supplementary figures and images for: MYEOV functions as an amplified competing endogenous RNA in promoting metastasis by activating TGF-β pathway in NSCLC
Source: Oncogene. 2018 Sep 4;38(6):896–912. doi: 10.1038/s41388-018-0484-9 (PMC6756124; doi:10.1038/s41388-018-0484-9)

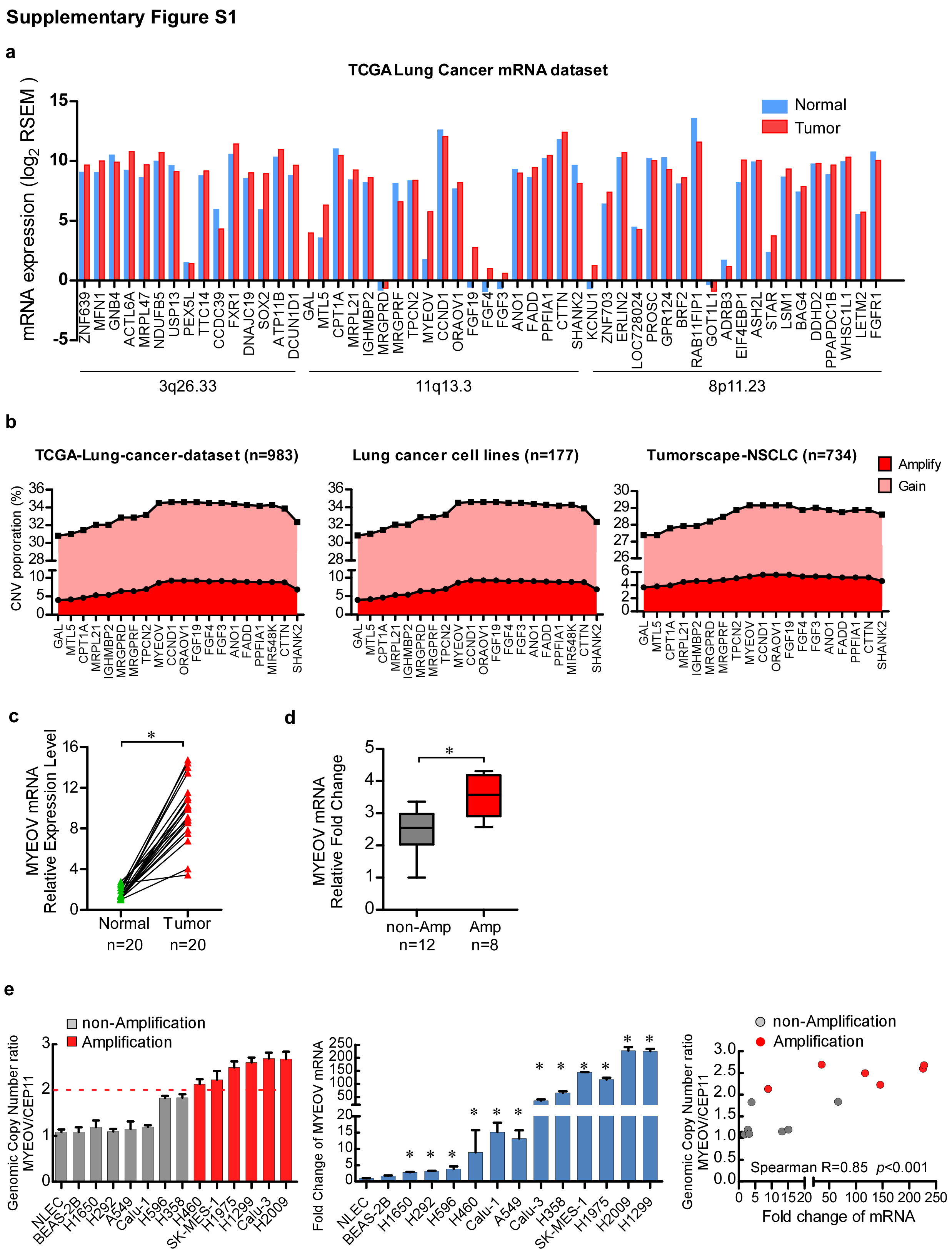

Supplement: Supplementary file 4 — Supplementary Figure 1 [file 41388_2018_484_MOESM4_ESM.tif]

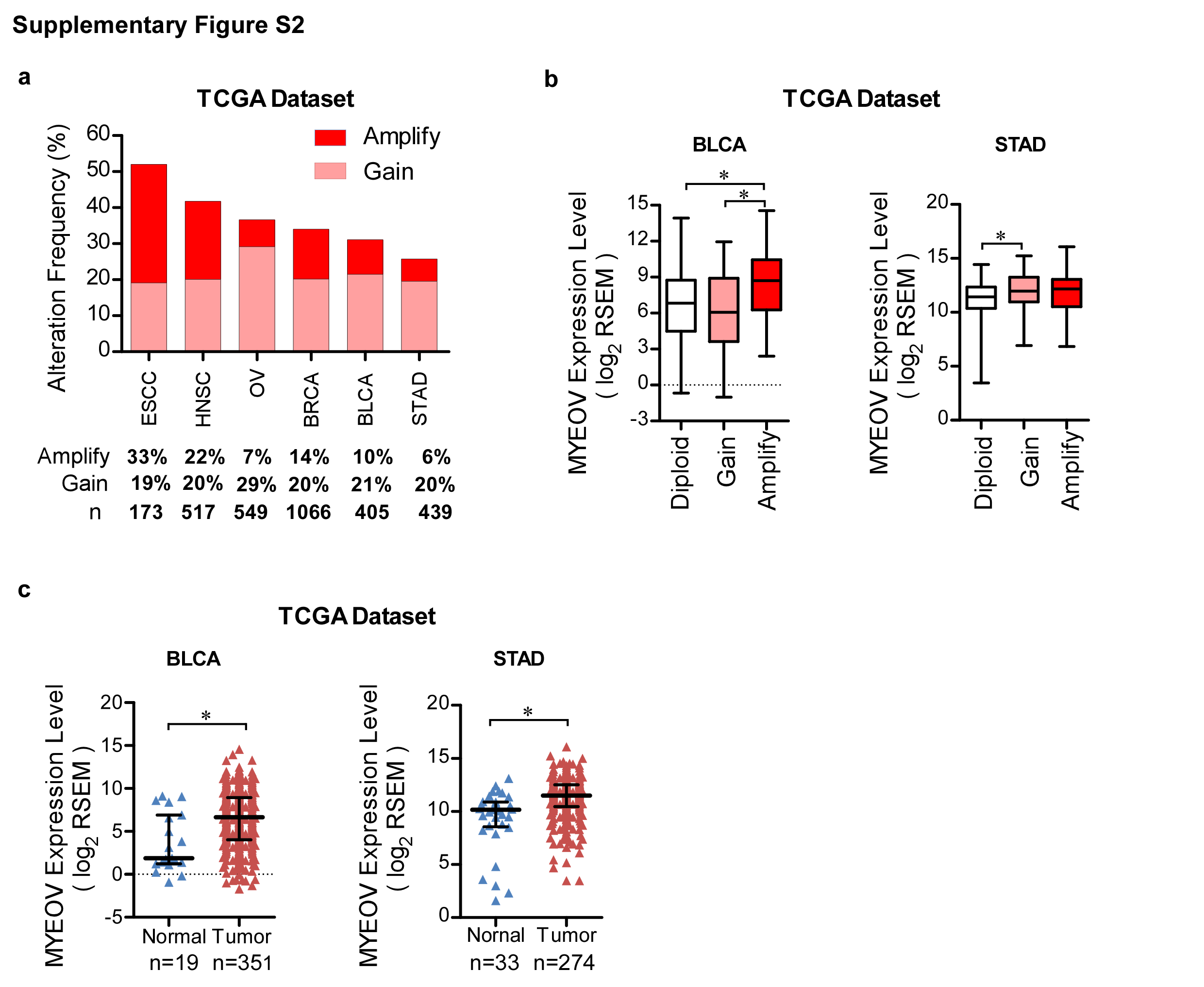

Supplement: Supplementary file 5 — Supplementary Figure 2 [file 41388_2018_484_MOESM5_ESM.tif]

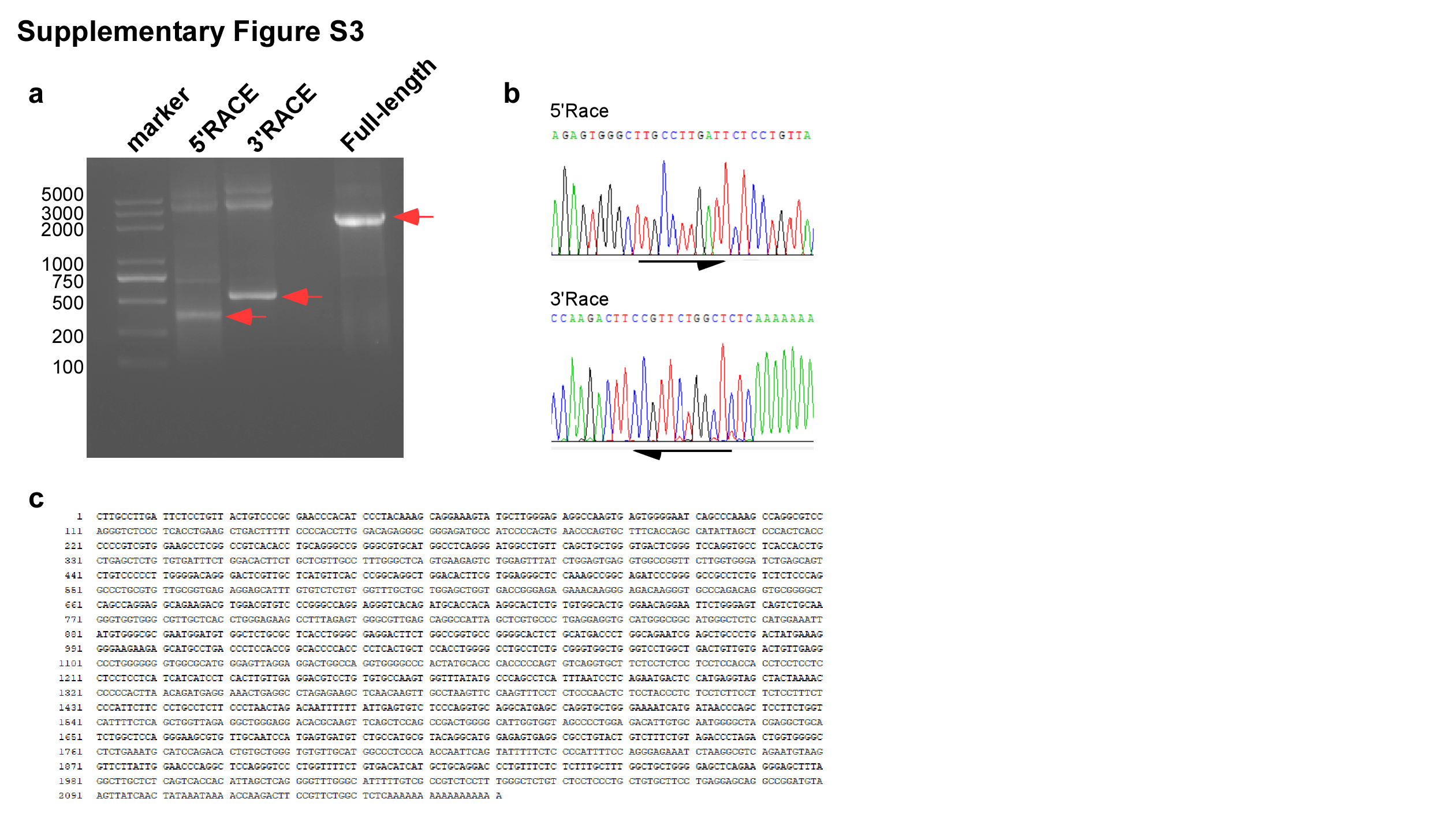

Supplement: Supplementary file 6 — Supplementary Figure 3 [file 41388_2018_484_MOESM6_ESM.tif]

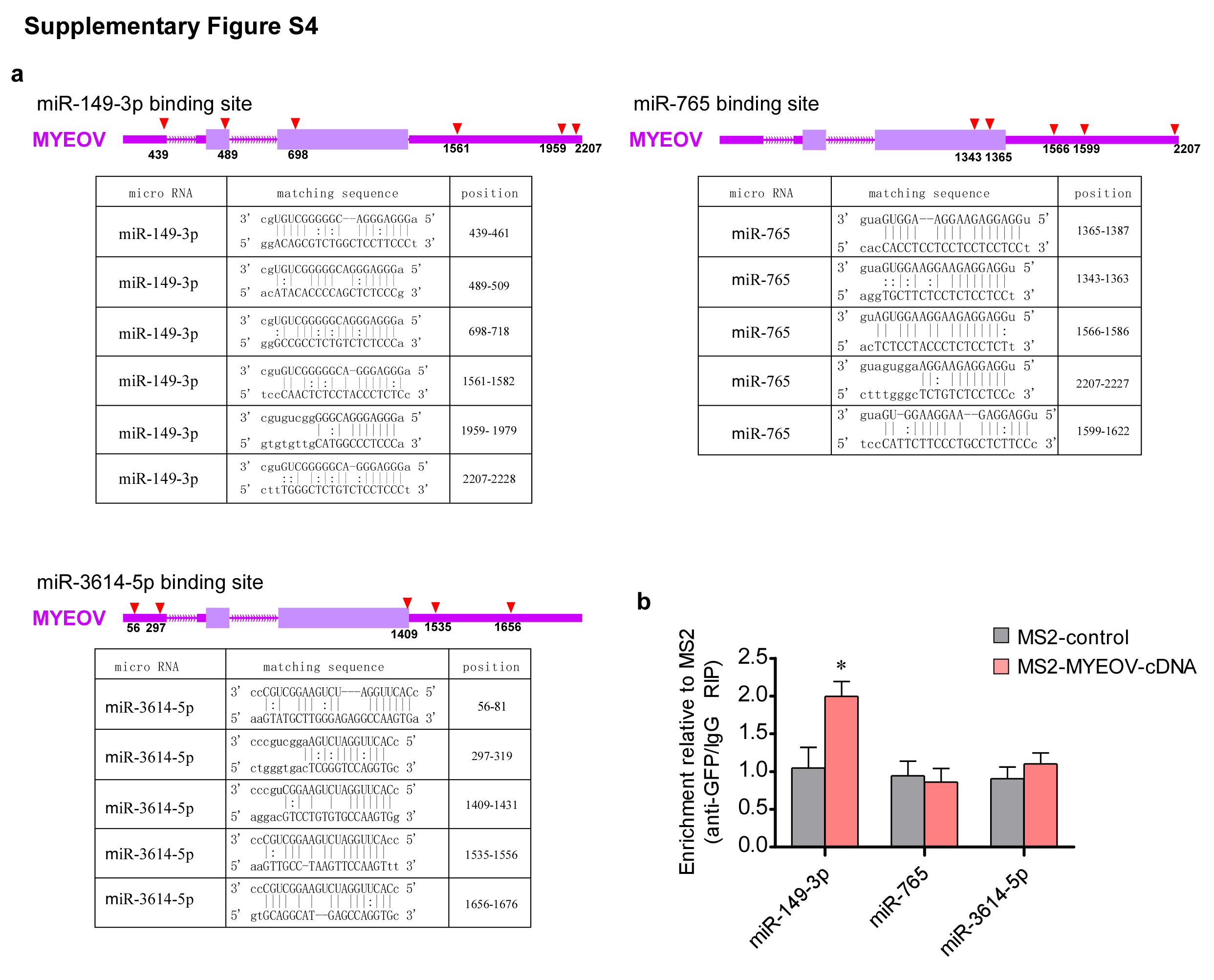

Supplement: Supplementary file 7 — Supplementary Figure 4 [file 41388_2018_484_MOESM7_ESM.tif]

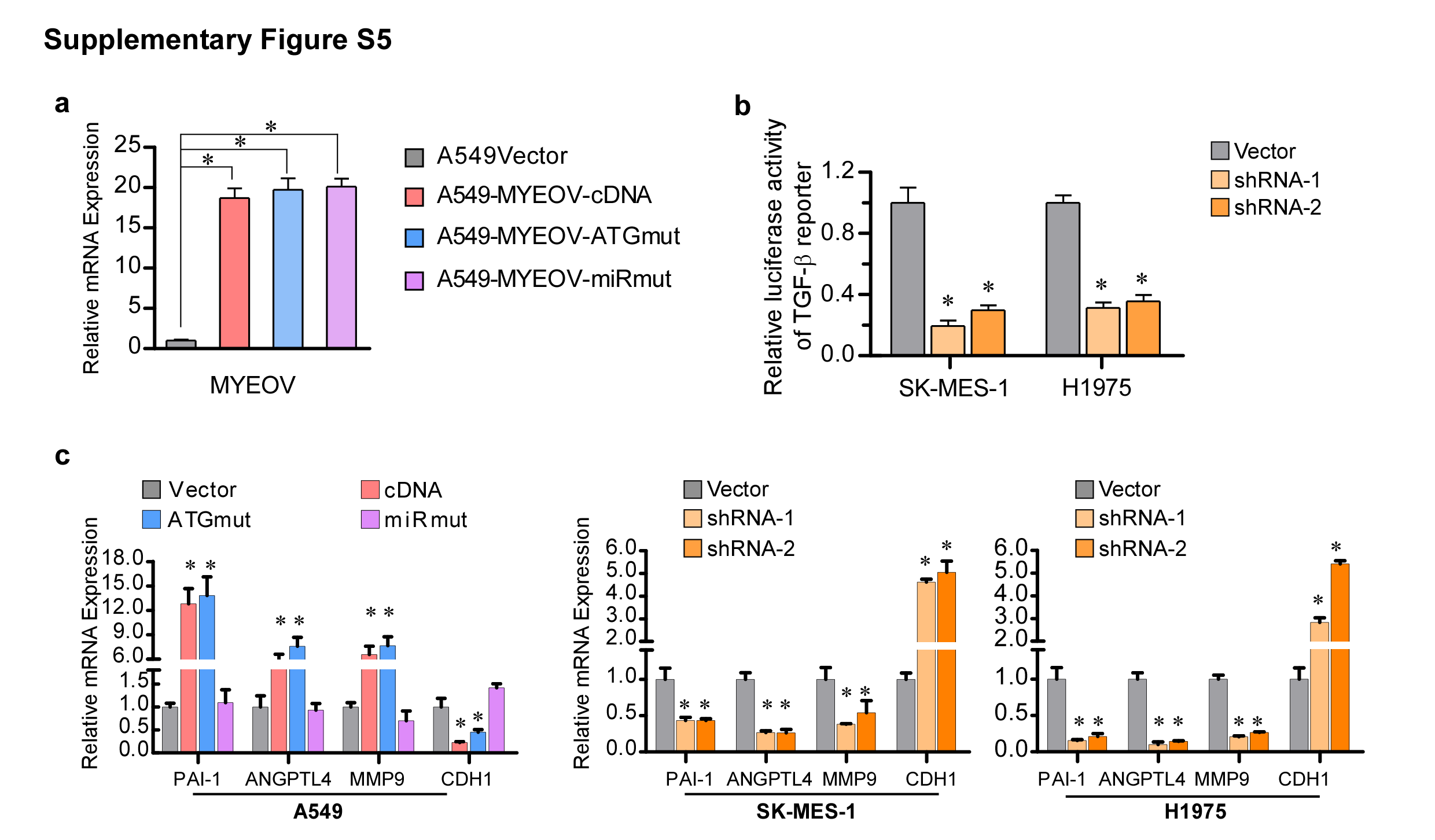

Supplement: Supplementary file 8 — Supplementary Figure 5 [file 41388_2018_484_MOESM8_ESM.tif]

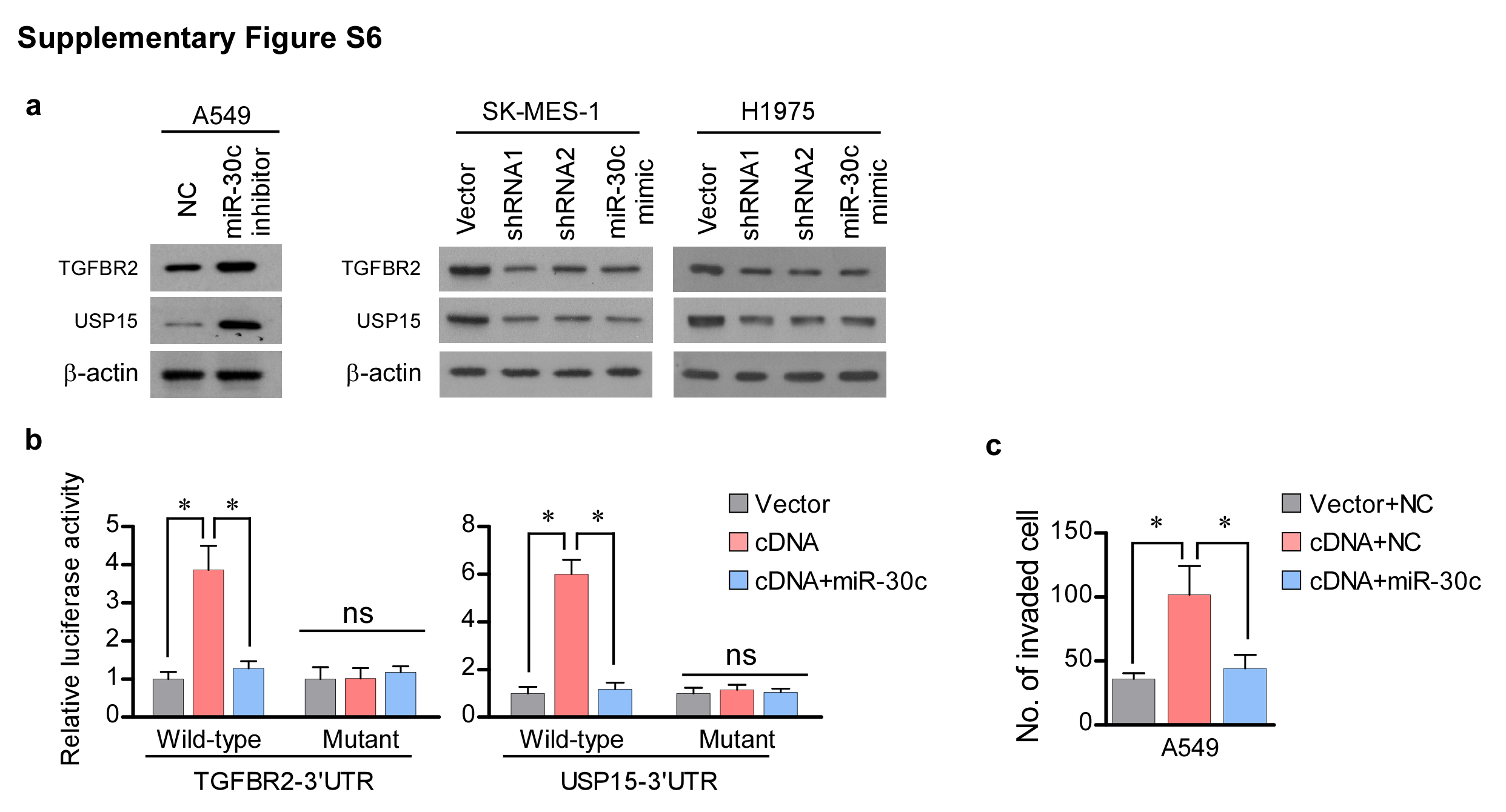

Supplement: Supplementary file 9 — Supplementary Figure 6 [file 41388_2018_484_MOESM9_ESM.tif]
